# Perceptions and Experiences of Health Care Workers on Accountability Mechanisms for Enhancing Quality Improvement in the Delivery of Maternal Newborns and Child Health Services in Mkuranga, Tanzania

**DOI:** 10.3389/fgwh.2022.868502

**Published:** 2022-06-30

**Authors:** Francis August, Tumaini Nyamhanga, Deodatus Kakoko, Sirili Nathanaeli, Gasto Frumence

**Affiliations:** ^1^Department of Development Studies, School of Public Health and Social Sciences, Muhimbili University of Health and Allied Sciences, Dar es Salaam, Tanzania; ^2^Department of Behavioral Sciences, School of Public Health and Social Sciences, Muhimbili University of Health and Allied Sciences, Dar es Salaam, Tanzania

**Keywords:** accountability mechanisms, maternal health, perceptions, experiences, health care workers

## Abstract

**Background:**

Maternal mortality estimates globally show that by 2017 there were still 211 deaths per 100,000 live births; more strikingly, 99% of them happen in low and middle-income countries, including Tanzania. There has been insufficient progress in improving maternal and newborn health despite the efforts to strengthen the health systems, to improve the quality of maternal health in terms of training and deploying human resources for health, constructing health facilities, and supplying medical products. However, fewer efforts are invested in enhancing accountability toward the improvement of the quality of maternal health care. This the study was conducted to explore the perceptions of healthcare workers regarding accountability mechanisms for enhancing quality improvement in the delivery of maternal newborn and child health services in Tanzania.

**Methods:**

We adopted phenomenology as a study design to understand how health workers perceive accountability and data were collected using semi-structured interviews. We then used thematic analysis to analyze themes and sub- themes.

**Results:**

The study revealed four categories of perceptions namely, differences in the conceptualization of accountability and accountability mechanisms, varied opinions about the existing accountability mechanisms, perceived the usefulness of accountability mechanisms, together with perceived challenges in the enforcement of accountability mechanisms.

**Conclusion:**

Perceived variations in the understanding of accountability among healthcare workers signaled a proper but fragmented understanding of accountability in maternal care. Accountability mechanisms are perceived to be useful for enhancing hard work in the provision of maternal health services. Moreover, inadequate motivation resulting from health system bottlenecks tend to constrain enforcement of accountability in the provision of maternal care services. Thus, we recommend that the government should deal with health system constraints and enforce regular monitoring and supervision.

## Introduction

Global maternal mortality estimates by UNFPA in 2017 show 295,000 maternal deaths, the ratio is 211 deaths per 100,000, live births (1), and more strikingly 99% of these deaths occur in low and middle-income countries (1). The African continent, mainly the sub-Saharan the region has added to 66% of all maternal mortality trends in 2015 (2). In Tanzania, signs of declining the trend of maternal mortality was recorded between 2005 and 2014 from 578 to 432 per 100,000 live births (3). However, maternal mortality ratios remained high and unchanged within a wide range of confidence intervals on the estimates of 556 per 100,000 live births in 2016 (4, 5). There has been insufficient progress in improving maternal and newborn health globally as the Millennium Development Goals (MDGs) four and five were not achieved by 2015. Not much improvement was achieved in reducing maternal morbidity and mortality (6, 7). Similarly, Tanzania was not able to meet the expected MDG target until 2015 (5, 8). This persistence of high levels of Maternal Mortality Ratios (MMR) have persisted despite the investment of resources to improve the quality of maternal and newborn care in the areas of training and deployment of human resources for health, construction of health facilities, and supply of medical products (9–12).

However, relatively fewer efforts have been put into enhancing accountability toward improving maternal and newborn care quality. Responsibility, which is an integral component of the governance and stewardship building block of the health system is barely emphasized in health facilities, as a mechanism for enforcing better results in the delivery of maternal and child health services (13, 14). The other blocks include human resources for health (HRH), health financing, medical products, service provision, and health information system (HIS) (15). In the health systems, context accountability implied that a governing body that is legitimate and has authority is in a position to make mandate subjects or organizations to attain specific objectives. The subordinates must account for their achievements under the goals (16). In the hospital setting, accountability is traced at three levels (Health Managers/in-charges, service providers, and patients or clients). In these three levels, there is either a line of command, enforcement, performance, or feedback, which is part of the patients' level. Since this conception draws from a human rights perspective, citizens who are the right holders, expect health workers' services to be good. At the same time, in-charges in the hospital are government custodians entitled to ensure equitable health services. Each part is obliged to play its part, and where there is a shortfall, each part ought to be answerable. For this study, accountability mechanisms are governance tools that seek to regulate answerability between the health system and the community or between different levels of the health systems such as the hospital management and the health care workers who are subordinates (17). Also, health care workers are referred to as those working in a hospital setting who help mothers and newborns to survive; such as nurses, midwives, and laboratory scientists.

Healthcare personnel are critical players in the delivery of quality maternal and newborn care. Little is known about how accountability is perceived by health care workers in the delivery of maternal newborn and child health services. Thus, understanding the perceptions of health care workers regarding accountability mechanisms will make it easier to comprehend how they perceive them, make sense of them, interpret them, attach meaning to them, and what it takes to enforce accountability more smoothly. Furthermore, exploring the perceptions of health care workers will add value in the ongoing implementation of health systems interventions geared toward improving the quality of care in resource constraint settings. Some studies have documented the contribution of accountability mechanisms in enhancing the quality of care by highlighting that it is the lubricant of other health system domains, which enables them to work in a much more interconnected and interrelated (16–19) manner. This study aimed at exploring health workers' perceptions on accountability mechanisms instituted in health facilities for enhancing quality improvement in the delivery of maternal newborn and child health services (19–21).

## Methods

### Study Design

In this particular study, we used phenomenology (22) as an approach to understanding perceptions of health care workers regarding accountability mechanisms for enhancing the quality improvement in the delivery of maternal health care services. Phenomenology was used as an approach that seeks to describe the essence of a phenomenon (accountability) by exploring it from the perspective of those who experience it (health care workers) (23). This approach is useful for studying health workers' lived experiences within their context—health facility; in terms of what and how they experience it. The study seeks to describe the essence of the phenomenon of the perception of health care workers of accountability mechanisms. We explored the perspective of health care workers who have experienced it, make sense of the phenomenon and come up with their interpretations.

This approach also helps to explore the lived experiences of health care workers and how they manifest in their working conditions through consideration of selfhood, sociality, embodiment, temporality, and spatiality (22, 23). By this approach, we see lived experiences as an interpretive process situated in a health workers' world. With the aid of this approach, we can best understand the perceptions of health workers on the enforcement of accountability mechanisms together with the meaning, they attach to them and their contribution to quality improvement in the delivery of maternal newborn and child health. We collected data from May to June 2019.

### Study Area

We conducted this study in Mkuranga District Hospital, located in the Pwani region in Tanzania in May and June 2019. The district also serves as the focal point where many maternal newborn and child health-related interventions are implemented; this includes “*Jiongeze Tuwavushe Salama*” an intervention which aimed to urge all health care workers working in the reproductive and child health section to work beyond their normal routine to help mothers to survive.

### Study Population and Recruitment of Study Participants

We recruited 25 participants ranging from nurses, midwives, and doctors working in the Reproductive and Child Health (RCH) and labor ward, and anesthesiologists to participate in our study. We purposively selected study participants in consultation with the in-charges of the section that provides RCH-related services. In each part of the hospital, we identified Key Informants with the help of the in charge of the particular section/unit to provide in-depth information about accountability issues that transpire in their daily responsibilities. Respondents involved in the study include nurses, midwives, doctors, clinical officers, laboratory scientists, and pharmacists. Their level of education ranged from certificate and diploma to degree holders. The age of the respondents ranged from 25 to 50 years. We conducted twenty-five Key Informant Interviews (KIIs) with health care workers including nurses and midwives working in both RCH and labor ward, doctors working in both RCH and labor ward. Key informants were purposively selected because they are directly involved in day-to-day reproductive and child issues at the district hospital. Henceforth, we conducted key informant interviews between May and June 2019. We used purposive sampling because only a limited number of informants could serve as the primary source of data. For instance, in this study, only healthcare workers providing RCH services were eligible for this study (24).

## Data Collection Techniques

### Key Informant Interviews

A semi-structured interview guide containing questions about the perceptions and experiences on accountability mechanisms, opinions of the existence of particular accountability mechanisms, the usefulness of accountability mechanisms, and the intervention measures for enforcing accountability mechanisms for enhancing quality improvement in the delivery of quality maternal health services were used. Probing questions were about how health care workers perceive specific accountability mechanisms if they are at all binding and what should be done to implement better accountability interventions geared for quality improvement. We developed the questions from the literature review.

### Data Collection Process

We conducted in-depth interviews with a research assistant who was trained and oriented to the study protocol. Before starting data collection, we pre-tested the interview guides in Kisarawe District Hospital is located in Pwani Region in Tanzania. We did it to ensure the quality of the responses emanating from the interview guide questions by adjusting items to ensure that data is adequate, appropriate, and precise. We approached Informants through the Matron in charge of nurses. Also, we contacted doctors through the medical officer-in-charge of the hospital. After identifying the participants, we made appointments based on their availability. On the appointment day, the first author, together with the research assistant, first explained the study details to familiarize study participants with the study before seeking their consent. In the course of conducting in-depth interviews, we probed participants to ensure that we got the actual meanings of the aspect of accountability in their context. We conducted interviews and discussions in Kiswahili and audio-recorded them. During the interviews, the research assistant took notes.

Interviews lasted for about 30–60 min and were carried out in offices identified by the interviewees. The venues were viable for sufficient privacy and confidentiality. The interviews were conducted by the first author and the research assistant. The saturation point was reached when there was no new information was coming out of the interviews (2).

### Validation Strategies

Validation strategies include prolonged engagement and persistent observation. The first author and the research assistant sought to build trust with participants, learning the culture of the working environment at the District hospital, and every evening after data collection, they went through all the audio recordings to check for completeness of the information and the extent to which the questions were asked appropriately.

Peer review or debriefing enhanced external checks of the research process. Some literature refers to this as “the devil's advocate” wherein our study, the primary supervisor together with other co-authors went through the transcripts to identify meaning and interpretations coming up to ensure validation of the data collected (25, 26).

### Data Analysis

All KIIs were transcribed verbatim and then translated into the English language before analysis. We employed a thematic analysis to develop themes that were coming up repeatedly. We involved six steps: familiarization with text/transcript, identification of codes (highlighting phrases related to accountability) that were emerging, looking at general themes, reviewing them, defining and naming them, and writing them up (27). We started the analysis by allowing all the authors to familiarize themselves with the data to understand accountability. The emerging codes were based on how accountability can contribute to enhanced quality improvement in the delivery of quality maternal and newborn health services (28). From the codes, we constructed Sub-themes to fit the context of answerability responsiveness, together with supervision/monitoring. Inter-coder reliability involves the extent to which two or more independent coders agree on how to code the content of qualitative data. In this study, the first author Francis August (FA), and the second author Tumaini Nyamhanga (TN), independently analyzed the data coming up with several codes through which we discussed and agreed on the coding content emanating from the transcripts.

### Ethical Considerations

We sought ethical clearance to undertake the study from the Muhimbili University of Health and Allied Sciences Institutional Review Board (IRB). We also sought and obtained informed consent from the participants by giving them consent forms to read, agree, and sign. We ensured that confidentiality, privacy, and anonymity were considered to protect the participants. We conducted interviews in suitable places that provided privacy and comfort for the participants. We excluded names and other possible identifiers from the data set to guarantee the anonymity of the study participants.

## Results

The study identified health workers' perceptions of accountability presented as themes and sub-themes emerging from the analysis. Four themes were identified, namely: perceived variations/differences in understanding accountability, accountability being useful for inculcating answerability, perception about the implementation of different accountability structures and mechanisms, and varying opinions about existing accountability mechanisms (see Table 1).

**Table 1:** Summary of the findings showing themes and sub-themes.

**SN**	**Themes**	**Sub-themes**
1	Perceived variations/differences in understanding accountability	Adherence to ethics and procedures, and sometimes legal aspects;
		Accountability mechanisms create fear among health workers;
		Accountability is regarded as awareness of one's duties and adherence to the job description.
2	Accountability is useful for inculcating answerability although sometimes it establishes a state of fear among healthcare workers	Health care worker's self-consciousness about answerability;
		Enforcement of accountability leads to the reduction of negligence;
		Accountability reinforces responsiveness among health care workers.
3	Varying opinions about existing accountability structures and mechanisms	An automated system of tracking attendance as an objective control of absenteeism;
		Ward rounds are vital for adherence and compliance checks;
		Meetings provide an avenue for implementing disciplinary measures and providing an avenue for reminding about and recollection of clinical practices;
		Maternal death audits are done as per government directives, and their outcomes may lead to the regular supply of medical equipment and human resources for health.
4	Perceived enforcement mechanisms of challenges to accountability	Enforcement of accountability is hampered by the inadequate deployment of human resources for health;
		Insufficient motivation among health care workers makes enforcement of accountability ineffective;
		Political interference in the provision of medical services compromises medical professionalism.

These themes and their respective sub-themes are presented in the sections that follow. A summary of the categories of accountability mechanisms (performance, managerial, and democratic) and their associated contributions to quality improvement are shown in Table 1. Some of the mentioned mechanisms include clinical meetings, biometric tracking attendance, maternal mortality audits (Open Performance Review and Appraisal System—OPRAS), ward rounds, regular supervision, and surprise visits by the health administrators. Most health care workers perceive accountability mechanisms as useful and essential and believe that they inculcate the spirit of answerability among healthcare workers, especially those providing maternal and child health services. However, it was found that without ensuring that hospitals are well-equipped with the required medical equipment, workers are well remunerated, and their demands are taken care of, it will be challenging to enforce accountability mechanisms.

### Theme 1. Perceived Variations in Understanding Accountability

We found that healthcare workers had different ways of understanding what accountability is. Respondents had varied definitions of accountability, ranging from awareness of one's duties, getting to work on time, avoiding the use of vulgar language toward patients, and answerability. These variations were captured in two sub-themes, as described in the sections that follow.

#### Sub-theme 1. Accountability Is Regarded as Awareness of One's Duties and Adherence to the Job Description

Respondents stated that accountability involves health care workers knowing what brings them to work every day. The respondents articulated this as self-awareness and performance of daily clinical duties. They emphasize the delivery of services following the terms of reference they signed during induction.

“*…. In my view, accountability starts with me; it is work that brings me here, to provide required services to patients as per my job description”. (Doctor RCH)*.

#### Sub-theme 2. Accountability Is Regarded as Adherence to Ethical Principles

Some healthcare workers were of the view that in the hospital setting, there are stipulated ethical principles that guide workers' actions, deeds, and motives, and they vehemently/strongly inform them of what is right or wrong. Being accountable in this understanding would mean abiding by the stipulated norms/codes of conduct. One of the respondents thus said:

“*… accountability refers to the system of working by abiding by ethics and legal procedures in helping mothers and babies survive. When you use vulgar language to a client, or when a maternal death has happened……, you are summoned by the authority and required to explain by writing a letter or being interrogated and reprimanded in a meeting with the hospital administrators” (Nurse-Midwife labour ward)*.

### Theme 2. Accountability Is Useful for Inculcating Answerability Although Sometimes It May Create a State of Fear Among Health Care Workers

Most the health care workers perceived accountability mechanisms as valuable and essential and believe that they inculcate the spirit of answerability among health care workers, especially those providing maternal and child health services. However, some were of the view that such accountability mechanisms may create fear among health workers. The usefulness of accountability mechanisms were captured in the following sub-themes.

#### Sub-theme 1. Health Care Worker's Self-Consciousness About Answerability

We learned from the participants that, given the maturity of a health care worker, their training, and the extent to which they sought the job, they are obliged to be accountable for working day and night to help mothers and babies survive. We were informed that healthcare workers are compelled to abide by the contract they signed when seeking employment, where they promised to fulfill all their obligations as required.

“*For me, accountability goes deeper than what a person has been assigned to do. and do it is diligently as required. You applied for the job and you got employed. You are supposed to do everything that you are obliged to do accurately, without complaints, and without harming the patient or yourself, and be able to deliver the best of your abilities in service provision” Medical Doctor in the labour ward*.

#### Sub-theme 2. Enforcement of Accountability Leads to the Reduction of Negligence

This concept came about as the state of working cautiously and being mindful of being asked to report what transpired while a health worker is on duty. It prompts a sense of answerability, which is equivalent to enhancing quality improvement in the provision of maternal health care services. It is contended that even in other sectors of one's work-life if one works while knowing that they will be answerable, it's likely that they will be more cautious and refrain from negligence in the course of providing maternal health services to avoid negative repercussions.

“*Accountability is beneficial because human beings are not perfect. Some people tend to transfer their home affairs to the working place and this entertains negligence; thus, being reprimanded when a mistake happens is useful in enhancing accountability in the course of health care provision”. Nurse in the labour ward*.

Another Key informant noted the same insisting that the aspect of answerability is central in accountability.

“*Whatever happens to the patient, it is the Doctor who is answerable. The nurse could be the second or the last person to be answerable” Doctor in the Labor ward*.

#### Sub-theme 3. Accountability Reinforces Responsiveness Among Health Care Workers

It was argued by the informants that accountability enhances responsiveness, which refers to the state of responding quickly to concerns of women coming to seek reproductive health services at the facility. One of the informants contended that, if accountability mechanisms are well implemented at the health facility, then health care providers are likely to act quickly and responsibly and save mothers' lives.

“*instituting accountability mechanisms in the context of health care provision helps to create fear of being reprimanded or terminated from work, which makes one fulfil their obligations, and accomplish their tasks on time. If I have made a certain mistake and I was called for interrogation and given a warning, I will make sure that I don't let it happen again'; Midwife labour ward*.

#### Sub-theme 4. Enforcement of Accountability Is Done on an *ad-hoc* Basis

Respondents believed that accountability is enforced through regular supervision and monitoring of service providers and health administrators. However, they cautioned that surveillance and monitoring are not regularly done unless there is an incident like a maternal death. Controls and inspections are not vigorously emphasized. This was well explained by one of the nurses working in the labor ward.

“*Follow-up and supervision should be done regularly regardless of the occurrence of events like maternal deaths. It happens that only when there is a maternal death then you see HMT members going around for inspection and supervision… this is not a good practice… we need to institute regular inspection and feedback mechanisms that can be used to monitor the performance of supervisors (the in-charges of the sections…the watchdog must also be watched!” Doctor in the labour ward*).

### Theme 3. Perceived Different Views About the Implementation of Different Accountability Structures and Mechanisms

The respondents expressed their varied opinions on the implementation of various accountability mechanisms that are enforced in the hospital setting to ensure quality improvement in the delivery of maternal health services. These include clinical meetings, biometric attendance tracking, maternal mortality audits, OPRAS, ward rounds, regular supervision, and surprise visits by health administrators. Perceptions of the existence of different accountability mechanisms were captured in the following sub-themes.

#### Sub-theme 1. Maternal Death Audits Are Usually Done as per Government Directives

Respondents pointed out that there is a committee, sometimes called the disciplinary the committee, that deals with all matters related to maternal and child deaths. We were informed that when a maternal death occurs, all those on duty are summoned to appear before the committee to explain what transpired that led to the death of the woman or child. In this case, they try to establish the cause of death, whether it happened out of negligence or because of other deficiencies, particularly unavailability of certain medical equipment or due to delays. The committee makes suggestions on what should be done, which may involve issuing a warning letter or ensuring a constant supply of the required equipment.

“*There is a team composed of all the in-charges of the hospital departments, i.e. the Matron, in charge of the labour ward, the in-charge of the RCH, and the in-charge of the pharmacy. The committee requires all those who were on duty to provide explanations to establish the cause of that particular death. This is in line with the policy of the Ministry of Health which indicates that when a maternal death occurs, it has to be audited within 24 hours. Thus when we receive a maternal death report or perinatal death, we respond very quickly and audit the death using our team, which consists of the matron, pharmacist, lab manager, Doctor, and the nurse in charge. We conduct an audit to establish the cause, particularly where the problem emerged from and identify a solution” (Doctor in charge of a unit)*.

Apart from indicating awareness of maternal death audits, the respondents indicated that the reviews are essential as they alert healthcare workers who are on duty that whenever a death occurs they will be answerable to higher authorities and they will face the consequences. Under such circumstances, medical errors are minimized.

“*Mostly when a death occurs, within two or three days, a report must be availed. A doctor must write a medical report detailing the events surrounding the death, what was the cause of death, who was on duty, etc. All those who were on duty are conscious that they will be asked to provide information about what transpired to cause the death, and then the report is forwarded to the district headquarters for documentation”. Doctor in RCH*.

#### Sub-theme 2. Maternal Death Audit Outcomes May Facilitate the Supply of Medical Equipment and Allocation of Human Resources for Health

We were informed that by performing maternal death audits, the hospital could document the causes of maternal deaths and come up with solutions for rectification, including increasing the deployment of health workers, ensuring a constant supply of medical equipment and the required medicines for the survival of mothers.

“*Regarding maternal death audits, when you are called to a hearing, you are supposed to explain how you received the mother and the services you provided to her… you also have to explain whether she had prolonged labour or fetal distress; then explain the measures you took as a nurse; that is, the way you helped the woman and the challenges that likely contributed to the maternal death” Nurse-midwife*.

#### Sub-theme 3. Meetings Provide an Avenue for Reminding About and Recollection of Clinical Practice

It was reported by the informants that there are meetings for enhancing accountability, which range from daily management meetings to weekly clinical meetings where in-charges of the RCH and labor ward of the hospital is obliged to convene. Matters emanating from events that transpired for the past 24 h or complaints are presented. In these meetings, the medical officer-in-charge has to probe reported complaints from clients and provide directives to the issues that emerge. There might be warnings directed to those who were seen to have misbehaved or re-allocation of health workers in different departments as a way of rectifying reported shortfalls.

“*I have been working here for the past two years, whenever a mistake/misconduct that led to maternal death or a complaint is reported, a meeting is convened by the hospital management to discuss the matter and come up with recommendations to solve the problem”. In the meeting, all those who were on duty when the incident happened are summoned to provide details/explanations. During the meetings, health care workers are reminded about the spirit of hard-working and accountability in general. Also, warnings are given to those who were responsible for the problem or complaint” Doctor in the labour ward*.

#### Sub-theme 4. Meetings Provide an Avenue for Implementing Disciplinary Measures

It was reported that whenever there are issues related to unprofessional conduct, the disciplinary committee convenes a meeting. In such committee meetings, the defaulters are interrogated and given warnings accordingly.

…*.“there is a disciplinary committee which is responsible for handling problems or complaints. When something happens, the health worker responsible is summoned to a disciplinary committee meeting to explain and respond to further interrogation, then they are either given instructions on what to do next time something happens or a warning if they are seen to be responsible for causing the problem. If the same mistake occurs again, they are transferred to another ward, e.g. the pediatric ward, for further monitoring” Doctor in the labour ward*.

It was argued that these meetings are catalysts for behavior change as they influence staff to be cautious of malpractice.

“*When a mistake happens, we convene short meetings and inquire from those who were on duty the aspects that have not been rectified, and we rectify them if need be. The in-charge of the section gives the defaulters a warning. But if the said behaviour persists, the Medical officer-in-charge is informed and if it is something to do with nursing, the nurse in charge or Matron is informed about it for further steps. If the practice does not change at that level, the Medical officer-in-charge is informed for further disciplinary action. When you raise concerns, people change, and I have witnessed people changing” Clinical officer*.

#### Sub-theme 5. Ward Rounds Are Vital for Adherence and Compliance Checks

It was reported by the informants that ward rounds are also part of accountability mechanisms. This is done daily by doctors who go around the wards, from bed to bed, checking on the hospitalized patients' progress. After the rounds, steps are taken to fill the identified gaps.

“*We do ward rounds. As doctors, we write prescriptions, but the nurses are the ones who perform or administer the treatment and other clinical procedures. The Doctor has to oversee and check to see if the treatment procedures and drug administration are done as prescribed. We also interview patients regarding their treatment progress. After the ward rounds, we write reports or recommendations from what we observe or hear from patients.” Doctor in the labour ward*.

#### Sub-theme 6. Perceived Dissatisfaction With OPRAS as Business as Usual

The respondents presented their opinions on the Open Performance Appraisal System as being among accountability mechanisms. They said they do fill in OPRAS forms which set objectives and targets to be achieved annually. Particular forms are filled out by all government employees. Promotions are done based on one's performance captured through the OPRAS forms. However, some health care workers were skeptical about the way OPRAS forms are handled; in the absence of serious follow-up, the assessment lacks objectivity. Indeed, it was asserted that some staff do not even bother to fill out the forms.

“*I am aware that we are supposed to fill OPRAS forms, but since I came here two years ago, I have never filled such forms. I always hear that we're supposed to fill them, but nobody makes a follow-up to countercheck” RCH nurse*.

#### Sub-theme 7. An Automated System of Tracking Attendance as an Objective Control of Absenteeism

The informants believed that the *automated system of tracking attendance is sound as it objectively controls absenteeism*. It was noted that the previous manual filling of attendance books lacked objectivity, as it was easy for health workers to fabricate arrival and exit times. As one of the respondents said:

“*Biometrics: the fingerprint machine that is used to track attendance helps a lot. Health workers are always coming to work on time as opposed to the previous system where we used to sign an attendance book; you could sign and indicate that you arrived at work at 7:30 am while you arrived there at 8:30 am! But this machine automatically records the real-time you arrived at work. This makes people more responsible for coming to work on time as it records the exact time one signs in and signs out” Nurse-midwife Labor ward*.

### Theme 4. Perceived Challenges to Enforcement of Accountability Mechanisms

Respondents indicated that while accountability is useful for enhancing staff performance, some constraints should be considered. These constraints were captured in three sub-themes, namely: inadequate deployment of human resources, insufficient motivation among health care workers, and frustration from political interference. The details about these factors are presented in the sections that follow.

#### Sub-theme 1. Enforcement of Accountability Is Hampered by the Inadequate Deployment of Human Resources for Health

The respondents felt that health administrators and staff face difficulties in enforcing accountability due to a shortage of human resources for health. In this manner, the healthcare personnel are critical players in the delivery of quality maternal and newborn care. Health workers are overburdened with multiple tasks and end up compromising the quality of their outputs.

“*It is challenging to administer treatment adequately because of the shortage of staff. You may find that there are only two nurses on duty, and they are supposed to serve more than three units, while at the same time there are more than 40 patients. Now, if there are only two nurses, one must do rounds in all wards while the other one stays at the maternity and labour ward, respectively… tasks become overwhelming and tedious for them, resulting in possible drug mal-administration, failure to infuse required patients with intravenous fluid, failure to do transfusions for patients who are supposed to receive such services, etc.” Doctor in the labour ward*.

#### Sub-theme 2. Inadequate Motivation Among Health Care Workers Makes Enforcement of Accountability Ineffective

Some healthcare workers argued that accountability mechanisms are less likely going to be compelling to make health workers more responsible if the government and other related stakeholders are not taking steps to deal with outstanding demands from healthcare workers. It has been noted that most healthcare workers, like many government employees, have outstanding requests for promotions and unpaid extra duty allowances. These tend to compromise efforts done by the hospital, administrators to enforce accountability because health workers are demoralized. This concern was well expressed by one of the participants:

“*Health care workers, especially those working with pregnant women, are supposed to be motivated. I have noticed that many health workers are demoralized because of their needs, like extra duty allowances have not been paid for years. The higher authorities have frozen promotions. we are demotivated we only work on humanitarian grounds!” Nurse-mid wife labour ward*.

#### Sub-theme 3. Political Interferences in the Medical Field Compromise Medical Professionalism

Respondents pointed out political interference, as a factor that frustrates health care workers. Politicians were blamed for interfering with health care workers' professional duties for political benefits while compromising the trust of the population in health care workers. Even though politicians represent citizens' concerns, the approach used is highly demoralizing to health care professionals. This tendency may demoralize the spirit of hard-working and compromise efforts to enforce accountability and hard work in the context of health system challenges like the inadequate allocation of human resources for health and insufficient supply of medical equipment.

“*The tendency for politicians to come and tell us what to do and reprimand us in front of our clients don't create a good image in the health care setting. Whenever politicians come to the health facility for inspection and find shortcomings, it is would be more beneficial to convene a separate meeting and brief us on what we have not been able to perform… rather than telling us off in front of the clients/patients. Politicians are using health facilities to acquire political fame at the expense of ruining medical professionalism.” RCH Doctor*.

## Discussion

The study came up with the following key themes; Perceived variations in understanding accountability—denoting that health workers have a firm but fragmented knowledge about accountability. Accountability mechanisms were reported as being useful in enhancing answerability in the delivery of maternal health care services. Health care workers believe that accountability mechanisms are tantamount to increased responsiveness, hardworking, and enforcement in the context of health care provision. However, healthcare workers cautioned about the health system constraints like inadequate human resources for health, inadequate supply of medical equipment, and welfare-related concerns that hinder the enforcement of accountability mechanisms for enhancing quality improvement in the delivery of maternal and newborn health services. The themes and sub-themes are explained in detail below. The findings in this study were informed by phenomenology as an approach that focuses on lived experiences of subjects of research. It follows that perceived variations in the conceptualization of accountability among the health care workers are evidence individuals perceive and interpret concepts differently. Even though health care workers went through relatively the same training system and worked under the same health system but they have gone through different lived experiences and they attach different meanings to those experiences (3–5).

Variations in understanding accountability mechanisms suggest that health workers have a proper but fragmented understanding of accountability, which reflects an inadequate focus on accountability during pre-service training and implies that during training, the concept of accountability was barely emphasized and was irregularly mentioned as part of professional ethics. Concerning phenomenology, the approach used in this study, although health care workers are working under the same health systems, they do experience it differently and assign varied meanings. This results from personal knowledge and the idea of subjectivity (6).

Other studies have indicated that in the Tanzanian health training curriculum, accountability issues are irregularly emphasized in highly compacted subjects like professional ethics and it is also barely touched upon in nursing studies and obstetrics and gynecology subjects. On that note, medical training suffers from the absence of a specific module on accountability (29, 30).

Other studies have reported similar findings that accountability had been variably understood as answerability as well as an obligation and enforcement (30, 31). This presents itself in two dimensions; on one hand, the accounting agency (the in-charges of sections) that impose penalties in terms of implementation, surveillance, monitoring, oversight, control, checks, and penalties to duty bearers—nurses, midwives, and doctors working in the RCH on the other hand. In this view, duty bearers who violate public duties receive restraints and punishment from accounting agencies/enforcers (32). Similarly, other authors have categorized the term accountability as “being called to account for one's actions”; duty bearers providing maternal health services are made responsive to the public (women seeking maternal health services) wishes. Other sources have shown that the term accountability is an ambiguous and contestable concept in a manner that accountability forms can be termed diagonal and horizontal, and involve administration, citizens, clients, and civil society. These forms cater to checks and balances (33, 34).

Accountability is useful for inculcating the spirit of answerability but sometimes creates fear among health workers. Most healthcare workers perceive accountability mechanisms as useful nd essential for inculcating the spirit of answerability, especially among those providing maternal and child health services. Appreciation of the usefulness of accountability is crucial for quality improvement for it shows recognition of existing hierarchical relationships in the fulfillment of roles and responsibilities. The existence of this attitude among health workers is vital for quality improvement in maternal health services. Literature shows a more or less similar view about the usefulness of accountability mechanisms: it inculcates the spirit of providing feedback, more accuracy in performance and appraisal, and reinforcement of performance improvement. In the context of maternal health services, interventions for activating accountability are essential in the language of quality improvement and reduction of maternal morbidity and mortality (35).

Also, this study found a similar finding to other related studies that accountability mechanisms create fear among health workers. If anxiety is geared toward refraining from being irresponsible or negligent while fulfilling one's duties and obligations that are good; nevertheless, if fear among health workers makes them passive and dormant to avoid making mistakes, the health system will paralyze. Almost similar views were presented by a study done in the Democratic Republic of Congo (DRC), which demonstrated how fear could create problems in health care provision (36).

*Additionally, answerability* has to do with the state of working mindfully/knowledgeable of being asked to report what transpired while a health worker is on duty. This prompts the sense of hard-working, which is equivalent to enhancing quality improvement in the provision of maternal health care services. It is contended that even in other sectors of life when someone works while knowing that they will be answerable, it's likely that they will be more cautious about working without any sense of answerability. Studies have shown gaps in answerability concerning generating information through the performance assessment and monitoring system, which is lacking in the district health facilities. Most of them suffer from inadequate mechanisms to gather information on causes of maternal mortality, referrals, injuries, and inadequate follow-up. These aspects tend to compromise enforcement to answerability due to the inability to coordinate existing monitoring systems as well as due to a shortage of appropriate indicators and inconsistencies in data collection (37, 38).

Furthermore, accountability reinforces responsiveness among health workers. This has to do with the state of responding quickly to the concerns of women who come to seek reproductive health services at the facility. One of the respondents contended that, if accountability mechanisms are well implemented at the health facility then health care providers are likely to act quickly and responsibly to save mothers' lives. Early and quick responses to mothers seeking maternal health services are vital in reducing maternal mortality. This argument follows from the fact that some reported maternal morbidity and mortality result from delays and negligence among health workers who do not quickly respond to mothers when they show up for maternal health services. Similar studies have looked at the extent and capability of the citizens to hold policymakers, politicians, and providers accountable and make them responsive to their needs. It also went further in assessing social accountability mechanisms through which women's concerns are expressed and responded to in healthcare settings (18, 36, 39).

### Enforcement of Accountability Is Done on an *ad-hoc* Basis

Respondents thought that accountability is enforced through regular supervision and monitoring for both service providers and health administrators. They, however, cautioned that surveillance and monitoring are not regularly done. Unless there is an incident like a maternal death, supervisions and inspections are not rigorously emphasized. Follow-up and guidance should be routinely done regardless of the occurrence of maternal mortality. There is a need to institute regular checks and feedback mechanisms that can be used to monitor the performance of supervisors. The findings reflect negligence in supervision on the part of enforcers (the in-charges), which compromises consistency in monitoring and tracking progress in maternal health service provision. A similar view was articulated by (30) that regular supervision and inspection are essential for enforcement and answerability.

On the one hand, subordinates are obliged to inform their in-charges of what they are doing and on the other hand, power-holders can impose penalties on the duty bearers who have committed misconduct. The author polished it by mentioning aspects like surveillance, monitoring, oversight, control, checks, and balances which are vital for spearheading constant supervision and monitoring.

Perceptions of the existence and functionality of different accountability mechanisms. The informants seemed to be aware of accountability mechanisms such as maternal death audits, OPRAS, ward rounds, and an automated system of tracking attendance. This finding is in line with the phenomenological approach in the sense that, within the interpretive epistemology, variations on how the phenomenon (accountability) is experienced differently by different individuals, although they might be working in one institution (4). Details of the function and usefulness of the different accountability mechanisms are elaborated as follows.

Maternal death audits provide a basis for punishment and ratifications for quality improvement.

Maternal death audits are necessary and useful because they alert health care workers who are on duty that whenever a death occurs they will be answerable or face consequences decided by higher authorities. It was noted that a disciplinary committee responsible for auditing maternal deaths handle all matters related to maternal and child mortality. If these committees are mandated to propose and effect consequences for non-compliant agents, then, quality improvement is likely to be observed; contrary to the recurring situation whereby existing committees simply make propositions that may not be very binding then the sense of “business as usual” prevails. Findings from other studies are in line with our results in a manner that maternal death reviews are potent tools for accountability as well as to monitor implementation and evaluate the effectiveness of health care, especially at the district level (19).

Besides, a study conducted in Nigeria showed that maternal death audits contribute significantly to quality improvement in the delivery of maternal health services. The reason is that they establish causes and circumstances related to maternal death occurrence, identify seriously complications including delays in performing caesarian sections, unavailability of magnesium sulfate, and lack of safe blood transfusion services, and therefore improve quality of care. These reviews aim to gather lessons learned from past experiences that can provide a sound evidence the base for making informed decisions, and synthesizing findings from maternal death reviews and other obstetric audits, which are geared toward identifying barriers to and facilitators of the provision of obstetric care (40). Furthermore, studies show that maternal death audits are useful for documenting the causes of maternal deaths (40–42).

### Maternal Death Audit Outcomes May Lead to a Supply of Medical Equipment and Human Resource for Health

We also found that the hospital can document the causes of maternal deaths and come up with solutions to rectify the situation, including among other things, increasing the deployment of health workers and ensuring a constant supply of medical equipment and required medicines to save mothers' lives. Maternal death audits are in line with the 2010 United Nations (UN) Global strategy for women's and Children's health. In contrast, there was an agreement to review and monitor progress in counting all deaths and identifying the contributing factors (16).

*Clinical meetings provide an avenue for conveying reminders and implementing disciplinary measures* that range from clinical meetings and daily management meetings where the in-charges of the RCH and labor wards of the hospital are obliged to convene meetings where matters emanate from events that transpired in the past 24 h or complaints are presented. In the said meetings, the medical officer-in-charge has to probe clients' reported claims and provide directives on the various issues raised. There might be warnings directed to those who misbehaved or re-allocation of health workers in different departments aimed at rectifying reported shortfalls. The importance of clinical meetings has also been emphasized by (43) as similar to stimulating changes that seeks to make quality a more explicit part of the health care system.

### Ward Rounds Are Vital for Monitoring Adherence and Compliance

These are done daily by doctors who go around the wards, checking the progress of the hospitalized patients. During ward rounds, doctors have to cross-check what was prescribed to patients and what was administered or performed by nurses. The practice is good for quality improvement. Nevertheless, other cadres of health workers do not do similar rounds to enhance the quality of their services. The findings of our study suggest that there is a need to adopt this practice. Other studies have shown that ward rounds provide an essential avenue for nurses and doctors to interact during oral discussion forums. In that manner, doctors would use nurses to supplement information and provide extra details to patient assessments (44).

### Perceived Dissatisfaction With OPRAS “Business as Usual”

The OPRAS is an integrated system that seeks to improve performance by setting individual goals, measuring goal achievement, and providing feedback (45). However, some health care workers were skeptical about the way OPRAS forms are handled and feel that they are handled in a manner that is less important without serious follow-ups and penalties against those who does not fill out the forms? Besides, OPRAS was seen as not adding any tangible benefit. The study found out that health workers were not working as intended due to the modalities of measuring performance and poor implementation of feedback mechanisms. The study suggests that even though this mechanism determines promotion criteria for public servants, there is a need to review it to become more user-friendly. The study by Songstad et al. (45), which was also conducted in Tanzania showed that health workers are reluctant to fill the OPRAS forms as they do not lead to any financial gain, and sometimes they do not provide meaningful feedback on performance.

### An Automated System of Tracking Attendance as External Control of Absenteeism Among Healthcare Workers Signaled to Be Effective

The informants were of the view that the systems help to expose dodgers during call hours. It was pointed out that the previous manual filling of the attendance books facilitated the fabrication of arrival and exit times. This positive opinion about the automated system of tracking the attendance of healthcare workers is an asset for enhancing staff compliance. The fifth phase The government of the United Republic of Tanzania invested heavily in the installation of biometric machines in many hospitals; as an intervention to enforce punctuality and eradicate cheating related to attendance at the workplace. Due to its effectiveness, this intervention needs to be implemented in all health facilities in the country.

Similarly, other studies have demonstrated the same importance (46), where the emphasis was put on the functionality of biometric machines beyond existing market offerings while improving modularity, extensibility, and the cost of ownership. It is, however, a costly and a monolithic investment that offers little customizability. This study suggests that while fingerprinting is useful in real-world settings, fewer efforts are needed to incentivize its usage over time.

### Perceived Challenges in the Enforcement of Accountability Mechanisms

We learned from the interviews that health staff thought that the enforcement of accountability mechanisms are affected by some factors such as the inadequate deployment of human resources for health and inadequate motivation among health care workers. Political interferences in medical professionalism and the inadequate supply of medical equipment tend to trigger limited quality improvement in healthcare. These findings suggest that the implementation of accountability mechanisms is not a simple undertaking if these constraints are not adequately addressed. This view is in line with other studies in Tanzania which showed that the country faces a critical shortage of human resources for health which tremendously affects the enforcement of accountability mechanisms and, consequently, compromise the quality of health services especially maternal and newborn child health services. Similar studies in sub-Saharan Africa (47–49) reported that health administrators face difficulties in enforcing accountability due to a shortage of human resources for health.

Furthermore, (49) political advocacy is necessary to increase citizens' voices in the provision of health services. Nonetheless, legislative advocacy is mistaken to involve the medical/health profession because civic virtues are said to be outside the medical professional realm. It is also argued that even if civic virtues were professionally mandatory, it remains unclear that political or public involvement is necessary for enforcing accountability in health care provision.

### Trustworthiness of the Research Findings

Trustworthiness in the phenomenological or interpretive study like this is a useful alternative for measuring the value of research and providing rigor in the research process. (4). In this context, therefore, we used five means of assessing the trustworthiness of qualitative research as propounded by Connelly (50), dependability, transferability, conformability, together with subjectivity and reflexivity. To ensure the credibility of the findings of this phenomenological study two aspects were considered, firstly the researchers were conversant with the subject matter of accountability in the maternal care and secondly, we ensured that trust was built among the researchers and interviewees such that they were able to engage in open and deep discourse about accountability. Also, to ensure the credibility of the methods used in this phenomenological study, the sample selected was appropriate to the research question under exploration.

This ensured authenticity and a comprehensive understanding of the phenomenon of accountability and thus increased the validity and reliability of the findings. To ensure dependability, we described in detail the overall study design where we used phenomenology that focuses on the lived experiences and meaning they assign to the concept of accountability and accountability mechanisms, data collection techniques, study the setting, study population, and data scrutiny. Trustworthiness of the findings was also in line with phenomenology as a design in research that seeks to affect change in the original setting, the transformation of the research informants as well as contribution to the realm of knowledge to shape the community. It also provided relevance to the research outcome and guide further recommendations in the epistemological contexts of the globe (4, 6, 7).

Confirmability, the use of phenomenology, allowed the researcher to explore the health workers' lived experiences and interpretations of the phenomenon of accountability as it appears to them in their working contexts. The themes in the study were inductively generated from the transcripts to ensure that they reflect the informant's perspectives, and themes were subsequently presented in sub-themes. A detailed description of the study setting, context, and data collection process and analysis ensured the transferability of the study findings.

Interviews were conducted by the first author (FA), a demographer with a Master of Arts degree in Demography and faculty at a reputable public medical training institution in Tanzania. They enhanced reassurance among the informants and thus facilitated the required cooperation. Furthermore, the familiarity of the researcher with health systems governance and its dynamics in the implementation of accountability mechanisms added credibility to this study. The researcher was supervised by three experienced health systems researchers, all with a background in health systems by training. Conducting interviews with nurses, midwives, and doctors may introduce bias and informants may over or under-report their perceptions. So, to reduce this bias, the first the author applied different interviewing techniques, including probing the same questions in different ways and acting naïve about the subject matter during the interview process. Besides, as a demographer and faculty teaching health systems and maternal health, with adequate comprehension of accountability mechanisms for enhancing quality improvement in maternal health, FA influenced the interpretation of data in some ways. Nevertheless, data analysis was done jointly with the second author (TN), and themes were generated through discussion and dialogue and collectively naming the themes.

Concerning subjectivity and reflexivity, the authors made assumptions about overt biases—during the research process. To reduce the extent to which FA's assumptions and bias could overly influence the research process, the authors had to understand several issues, including the process of self-awareness, which reflected FA's position as a researcher and the principal investigator as well as their personal experiences working in the health system. As non-clinicians who teach at the medical university, we had an external view of how the health system works and how accountability mechanisms are enforced. As researchers, we were eager to know-how accountability mechanisms are executed at health facilities from the insiders' point of view. With the scholarly consultations, discussions, and supervisory meetings, we could not be devoid of influences on the subject matter. Instead, it gave us more space for building trust in the health care workers that we interviewed and appreciating different views that emanated from the informants in their working contexts. Also, being familiar with the context of interviews was more conventional, uncovering vibrant information and delving into hidden aspects of accountability mechanisms. This process was supported by transparency, trust, and honesty from the health care workers. Furthermore, during the interview process probing was used to seek clarity and to check the accuracy of the information coming from the health care workers regarding accountability mechanisms.

Lastly, the involvement of the supervisors who are also co-authors but were not involved directly in designing the study helped to keep FA's views nuanced.

## Conclusions and Recommendations

Perceived variations in understanding accountability and related mechanisms signaled proper but fragmented understanding of accountability in the provision of maternal, newborn, and child health. Accountability mechanisms are vital for enhancing responsiveness, answerability, and enforcement for quality improvement in the provision of maternal, newborn, and child health but create fear among health workers. Health care workers perceive accountability mechanisms to be useful and essential for enhancing hard work in the course of providing maternal, newborn, and child health services. Still, they are enforced on an *ad-hoc* basis, and follow-up is only done in tragic situations.

Inadequate motivation resulting from delayed salary increments, unpaid extra-duty allowances, and promotions, tend to constrain efforts to inculcate the spirit of accountability in maternal, newborn, and child health services.

Health system constraints like inadequate deployment of human resources for health, an insufficient supply of medical equipment, and poor working conditions, among others, tend to water down the efforts put in by health facilities to enforce accountability in the provision of maternal, newborn, and child health services.

We recommend that curricula for health education should have a specific module or topic on accountability. It will teach uniformity in understanding the subject matter and will likely contribute to better performance and accountability in the provision of maternal health services. Health managers should conduct regular monitoring and supervision in the RCH and labor ward rather than wait until there is a maternal death. It will increase response preparedness among health care workers to save mothers in need of urgent maternal health services.

There is a need to introduce checks and balances in the provision of maternal health services; in other words, “*the watchdog must be watched”* and each unit must be under constant scrutiny. Findings from this study have shown that health workers are more likely to work cautiously when they know that an inspection is being done or that they will be asked to account for what they have done or not done, and how.

Responsible authorities should deal with health system bottlenecks that constrain the implementation or enforcement of accountability mechanisms. This is important because most health workers find it challenging to fulfill their responsibilities due to inadequate motivation, a limited supply of medical equipment, unfavorable working conditions, and unpaid extra-duty allowances.

Lastly, politicians should refrain from undue influence and intervention in the health profession. Although elected leaders are obligated to oversee the way health services are being provided, the current approach where politicians make surprise visits to facilities and intimidate the health care worker is contrary to the principle of autonomy and non-interference in the medical profession.

## Data Availability Statement

The raw data supporting the conclusions of this article will be made available by the authors, without undue reservation.

## Ethics Statement

The studies involving human participants were reviewed and approved by MUHAREC-Muhimbili University Research Ethics Committee. The patients/participants provided their written informed consent to participate in this study.

## Author Contributions

FA and TN designed and executed the research study protocol and prepared the first draft of the manuscript. DK, GF, and SN reviewed and assisted in revising the manuscript. All authors read and approved the final draft of the paper.

## Funding

SIDA—Swedish International Development Agency paid all expenses.

## Conflict of Interest

The authors declare that the research was conducted in the absence of any commercial or financial relationships that could be construed as a potential conflict of interest.

## Publisher's Note

All claims expressed in this article are solely those of the authors and do not necessarily represent those of their affiliated organizations, or those of the publisher, the editors and the reviewers. Any product that may be evaluated in this article, or claim that may be made by its manufacturer, is not guaranteed or endorsed by the publisher.
